# Effect of inorganic fillers on the light transmission through traditional or flowable resin-matrix composites for restorative dentistry

**DOI:** 10.1007/s00784-023-05189-7

**Published:** 2023-08-18

**Authors:** Rita Fidalgo-Pereira, Óscar Carvalho, Susana O. Catarino, Bruno Henriques, Orlanda Torres, Annabel Braem, Júlio C. M. Souza

**Affiliations:** 1 Center for Interdisciplinary Research in Health (CIIS), Faculty of Dental Medicine (FMD), Universidade Católica Portuguesa (UCP), 3504-505 Viseu, Portugal; 2 University Institute of Health Sciences (IUCS), CESPU, 4585-116 Gandra PRD, Portugal; 3 Center for MicroElectroMechanical Systems (CMEMS-UMINHO), University of Minho, 4800-058 Guimarães, Portugal; 4 LABBELS Associate Laboratory, University of Minho, Guimarães, 4710-057 Braga, Portugal; 5 Ceramic and Composite Materials Research Group (CERMAT), Department of Mechanical Engineering (EMC), Federal University of Santa Catarina (UFSC), SC 88040-900 Florianopolis, Brazil; 6 Oral Pathology and Rehabilitation Research Unit (UNIPRO), University Institute of Health Sciences (IUCS), CESPU, 4585-116 Gandra, Portugal; 7 Department of Materials Engineering (MTM), Biomaterials and Tissue Engineering Research Group, KU Leuven, 3000 Leuven, Belgium

**Keywords:** Resin-matrix composite, Light curing, Polymerization, Degree of conversion, Light transmittance, Fillers, Inorganic particles

## Abstract

**Objectives:**

The aim of this in vitro study was to evaluate the light transmission through five different resin-matrix composites regarding the inorganic filler content.

**Methods:**

Resin-matrix composite disc-shaped specimens were prepared on glass molds. Three traditional resin-matrix composites contained inorganic fillers at 74, 80, and 89 wt. % while two flowable composites revealed 60 and 62.5 wt. % inorganic fillers. Light transmission through the resin-matrix composites was assessed using a spectrophotometer with an integrated monochromator before and after light curing for 10, 20, or 40s. Elastic modulus and nanohardness were evaluated through nanoindentation’s tests, while Vicker’s hardness was measured by micro-hardness assessment. Chemical analyses were performed by FTIR and EDS, while microstructural analysis was conducted by optical microscopy and scanning electron microscopy. Data were evaluated using two-way ANOVA and Tukey’s test (*p* < 0.05).

**Results:**

After polymerization, optical transmittance increased for all specimens above 650-nm wavelength irradiation since higher light exposure time leads to increased light transmittance. At 20- or 40-s irradiation, similar light transmittance was recorded for resin composites with 60, 62, 74, or 78–80 wt. % inorganic fillers. The lowest light transmittance was recorded for a resin-matrix composite reinforced with 89 wt. % inorganic fillers. Thus, the size of inorganic fillers ranged from nano- up to micro-scale dimensions and the high content of micro-scale inorganic particles can change the light pathway and decrease the light transmittance through the materials. At 850-nm wavelength, the average ratio between polymerized and non-polymerized specimens increased by 1.6 times for the resin composite with 89 wt. % fillers, while the composites with 60 wt. % fillers revealed an increased ratio by 3.5 times higher than that recorded at 600-nm wavelength. High mean values of elastic modulus, nano-hardness, and micro-hardness were recorded for the resin-matrix composites with the highest inorganic content.

**Conclusions:**

A high content of inorganic fillers at 89 wt.% decreased the light transmission through resin-matrix composites. However, certain types of fillers do not interfere on the light transmission, maintaining an optimal polymerization and the physical properties of the resin-matrix composites.

**Clinical significance:**

The type and content of inorganic fillers in the chemical composition of resin-matrix composites do affect their polymerization mode. As a consequence, the clinical performance of resin-matrix composites can be compromised, leading to variable physical properties and degradation.

**Graphical Abstract:**

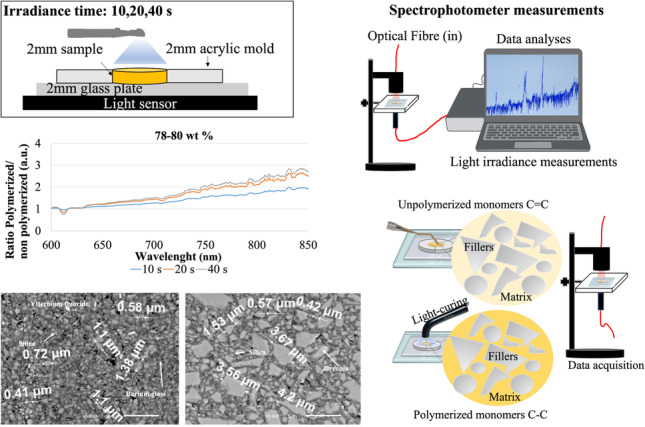

## Introduction

In the last decades, resin-matrix composites have become the most suitable materials in restorative dentistry due to their technological development on the chemical composition and processing. Nowadays, resin-matrix composites are used for indirect and direct restorations in restorative dentistry [1–3]. The chemical composition involving the inorganic fillers and polymeric matrix of the resin-matrix composites is a major factor that determines their optical and mechanical properties [2, 3]. However, the chemical composition and physical properties of the currently available resin-matrix composites widely vary from manufacturers and types of materials [4–6]. Inorganic fillers are added in the chemical composition of the resin-matrix composites to increase their mechanical properties and to mimicking the optical properties of enamel and dentin [7–9]. On light curing procedures, the inorganic fillers must allow the transmission of visible light required for the activation of the polymerization reaction of the polymeric matrix. Nevertheless, the polymerization can vary depending on the content, type, and size of inorganic fillers considering optimal conditions on the procedure and equipment of light curing procedures [9–11]. A lack of polymerization decreases the strength, elastic modulus, hardness, and wear resistance of the resin-matrix composites leading to the degradation and release of monomers to the surrounding tissues [12–15].

A resin-matrix composite comprises inorganic vitreous fillers dispersed in an organic matrix. The organic matrix involves a cross-linking of dimethacrylate monomers such as bisphenol A-glycidyl dimethacrylate (Bis-GMA), triethylene glycol dimethacrylate (TEGDMA), urethane dimethacrylate (UDMA), and ethoxylated bisphenol A dimethacrylate (Bis-EMA) [16–18]. Such combination of molecules results in an organic matrix that depends on the structure of the monomers and the polymerization reaction. On visible-light polymerization, camphorquinone (CQ) combined with a tertiary amine are incorporated in resin-matrix composites as a photoinitiator system [19–22]. Also, acyl and bisacyl phosphine oxide initiators can be utilized as single-component visible-light alpha-cleavage initiators at wavelengths usually below 450 nm [20, 23, 24]. In the organic matrix, CQ is stimulated by visible light irradiation in the range between 420 and 490 nm [15, 25, 26]. Commercially available resin-matrix composites show a weight percentage (wt. %) of inorganic filler content ranging from 40 up to 90 wt. % [7, 27]. A mixture of different inorganic fillers (i.e., glass ceramics and silica) at different sizes can be found in the chemical composition of current resin-matrix composites [6, 8, 28]. Nano- and micro-scale particles are combined in the resin-matrix composite microstructure to promote a mechanical reinforcement under chewing loading [6, 9, 28]. In fact, a high content of nano- and micro-scale particles results in a low organic matrix volume under polymerization. Indeed, inorganic fillers affect the polymerization shrinkage, wear, surface roughness, translucency, opalescence, and fluorescence of resin-matrix composites [18, 29, 30]. Resin-matrix composites with volume fraction up to 60 wt. % revealed increased values of flexural strength and elastic modulus [31]. Despite the influence of the inorganic filler content on the mechanical properties of resin-matrix composites, the nature and shape of the inorganic particles must also be considered [33, 34]. Spherical-shaped inorganic particles allow higher amount of fillers within the resin-matrix composites, and the enhancement of materials' strength comparatively to the materials with irregular filler particles, once the stresses tend to accumulate in the protuberances of the irregular-shaped particles [32]. Nevertheless, the effects of the content, size, and shape of inorganic fillers on the light transmission through resin-matrix composites are not entirely elucidated in literature.

The polymerization of resin-matrix composites for direct restorations can be achieved by demand using light-curing units (LCU) as a source of light ranging from 360 up to 500 nm [25, 35, 36]. Currently, light-emitting diodes (LED) are the most typical light source within wavelength ranging from 360 up to 500 nm, light irradiance between 400 and 1765 mW/cm^2^, and light exposure from 20 to 60 s [37–39]. The time of light exposure depends on the light irradiance as well as type and thickness of the restorative materials  to reach the energy required for the polymerization of the resin-matrix composite. The incident light can be reflected, refracted, absorved, scattered, and/or transmitted towards the material. On the resin-matrix composites’ surfaces, part of the light is reflected, due to the differences in the material refractive indexes [9]. Scattering also occurs when light passes through the material due to the existence of fillers and defects such pores or cracks. Scattering changes with the wavelength of incident light and is mostly determined by the particle size and by the relationship between their refractive indexes [9, 40, 41]. The accomplishment of the polymerization of the organic matrix leads to densely crosslinked, glassy polymer networks that provide high values of physical properties such as strength, elastic modulus, hardness, toughness, and wear resistance [42–44]. The optical and mechanical behavior of resin-matrix composites is strongly influenced by the degree of conversion (DC) of the organic matrix [14, 17]. DC percentage of resin-matrix composites is determined by a comparison of the peak height absorbance intensity of aliphatic carbon–carbon double bond (C = C) with aromatic C = C, before and after irradiation [18, 45]. Manufacturers recommend resin-matrix composite increments with 2-mm thickness [46, 47], to achieve adequate DC proportion. The clinical success of direct restorations depends on the light transmission, polymerization, and material properties [4, 5].

The relationship among light, polymerization, DC, and fillers raised the question on the influence of inorganic fillers content on light transmission through resin-matrix composites. Thus, the purpose in this study was to evaluate the light transmission through five different resin-matrix composites regarding the inorganic filler content. The following hypotheses were established: (i) the percentage of inorganic fillers has a significant influence on the light-curing transmission through the resin-matrix composites; (ii) the increase in the inorganic fillers’ amount induces a high light-curing transmission through the material.

## Materials and methods

### Preparation of specimens

**Fig. 1:**
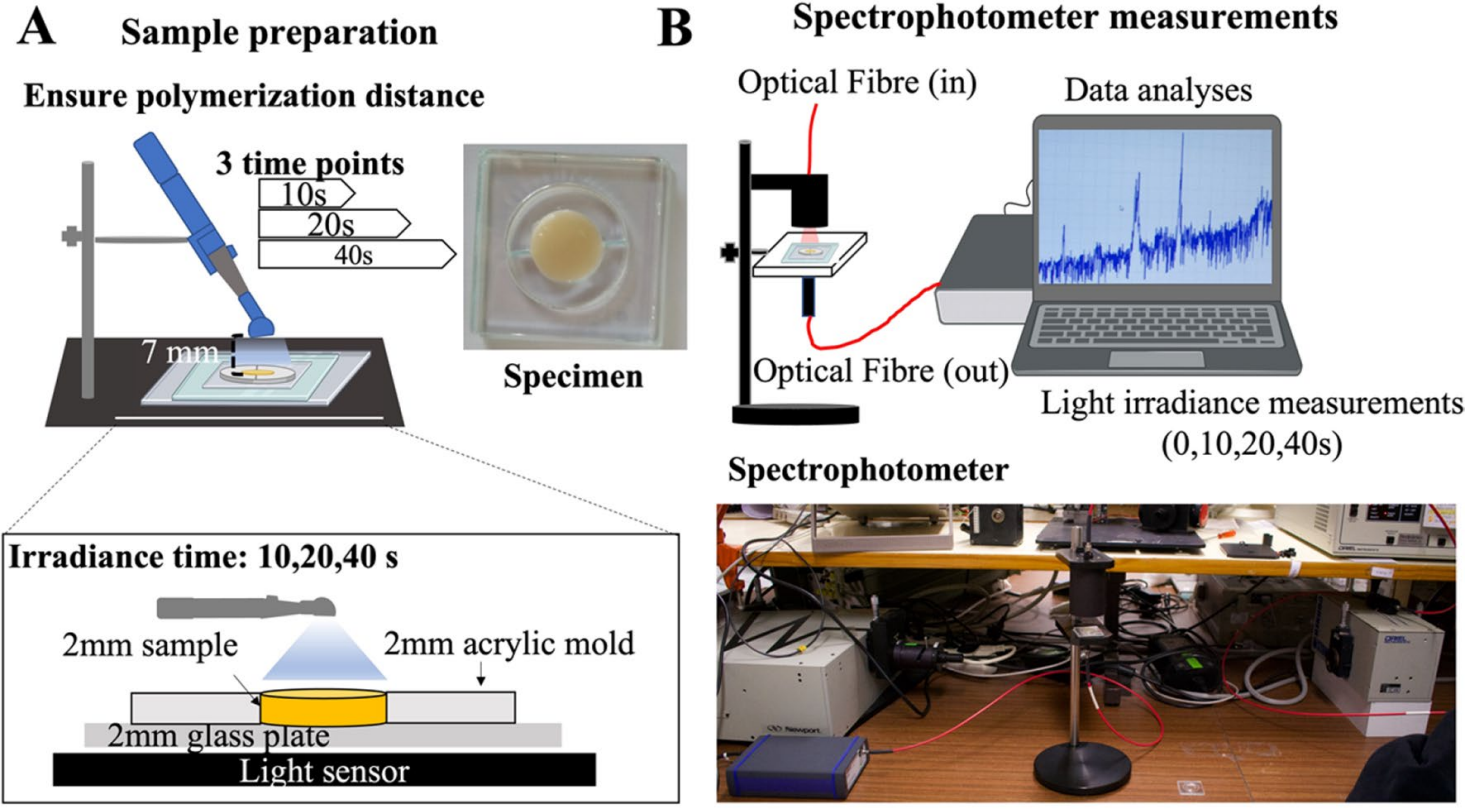
**A** Preparation of specimens and light irradiance time points. **B** Optical transmittance measurements

Disc-shaped specimens (diameter at 10 mm × thickness of 2 mm) were prepared from five different commercially available resin-matrix composites. Traditional and flowable resin-matrix composites (shade A1) were selected considering a variation proportion of the inorganic fillers’ content ranging from 60 up to 89 wt. %. The chemical composition of the resin-matrix composites was provided by the manufacturers, as seen in Table 1. Data of the resin-matrix composites were given by the manufacturers. Seventy-five specimens were prepared in a custom-made glass holder following the standard incremental technique, as seen in Fig. 1. Five specimens were prepared for each set of polymerization time point.

**Table 1:** The chemical composition of the tested resin-matrix composites provided by the manufacturers.

Material, Brand (manufacturer, country)	Organic matrix	Inorganic fillers (wt. %)	Filler shape, type, and size
Nano-hybrid resin-matrix composite, Grandio SO^TM^ ( VOCO GmbH, Germany)	Bis-GMA, Bis-EMA, TEGDMA, CQ	89	Silanized silicon dioxide nanoparticles (20–40 nm) and zirconia glass fillers (1 μm)
Nano-hybrid resin-matrix composite, Ceram × Spectra HV^TM^ ( Dentsply Sirona, USA)	Bis-EMA, UDMA, TEGDMA, CQ	78–80	Silanized barium glass, sphereTEC™ prepolymerized fillers, and non-agglomerated ytterbium fluoride; methacrylic polysiloxane nanoparticles. Fillers ranged from 0.1 to 3 µm
Sub-micron hybrid resin-matrix composite, Coltene Everglow^TM^ (Coltene, Switzerland)	Bis-GMA, TEGDMA, Bis-EMA, CQ	74	Amorphous silica, glass–ceramic, zinc oxide. Fillers ranged from 0.02 up to 1.5 μm
Flowable resin-matrix composite, Ceram × Spectra flow^TM^ ( Dentsply Sirona, USA)	UDMA, Bis-Hema, CQ	62.5	Barium- aluminum-borosilicate glass, ytterbium fluoride, iron oxide, and titanium oxide SphereTEC™. Fillers ranged from 0.1 up to 3.0 µm
Flowable resin-matrix composite, Coltene Everglow flow^TM^ (Coltene, Switzerland)	TEGDMA, Bis-GMA, CQ	60	Amorphous silica and zinc oxide. Fillers ranged from 0.02 up to 1.5 μm

*Bis-GMA*, bisphenol A-glycidyl dimethacrylate; *TEGDMA*, triethylene glycol dimethacrylate; *UDMA*, urethane dimethacrylate; *Bis-EMA*, ethoxylated bisphenol A dimethacrylate; *CQ*, camphorquinone

The polymerization of the resin-matrix composites was carried out using a LED light curing unit (Cordless LED light B™, Woodpecker Co., Poland). Light-curing unit (LCU) emitted light with a wavelength ranging from 430 up to 480 nm. Prior to the light-curing procedure, the irradiance of the device was measured by a calibrated radiometer (ProclinicExpert™, Montellano, Lisbon), showing systematically a steady irradiance at 350 mW/cm^2^. The LCU tip was mechanically positioned perpendicularly to the specimen surface plane and at 7 mm away from the surface, as seen in Fig. 1. The light irradiance was carried out following the guidelines from a previous study [44]. It should be emphasized that the light-curing procedure was performed in a controlled environment, with no expected light losses or dissipation through the surrounding environment.

### Optical transmittance tests

Optical transmittance measurements were performed for each specimen before and after light curing for 10, 20, or 40 s (Fig. 1A). Thus, the setup comprised the optical transmittance measurement of non-polymerized specimens, followed by the polymerization of the specimens, and then the optical transmittance measurement of light-cured specimens (Fig. 1B). The measurements were immediately performed after polymerization. For calibration purposes, optical reference measurements were acquired between each set of measurements. All the transmittance spectra were measured using a top-bench spectrophotometer with an integrated monochromator (AvaSpec-ULS2048XL EVO, Avantes, NS Apeldoorn, The Netherlands). The apparatus comprised a 200 W Quartz Tungsten Halogen light source (model 66,881, Oriel Newport, USA), optical-fiber probes, and a metallic holder for specimens (Fig. 1B).

The assays were performed for a wavelength range between 350 and 850 nm, with a measurement integration time of 10 ms. The integrated monochromator was coupled to a computer for data acquisition and smoothing, based on 100 measurements’ average (1 s total analysis time). The spectrophotometer has an optical bench with 37.5-, 50-, 75-, or 100-mm focal length, developed in a symmetrical Czerny-Turner design. Light enters the optical bench through a standard SMA-905 connector and is collimated by a spherical mirror. A plain grating diffracts the collimated light and a second spherical mirror focuses the resulting diffracted light. An image of the spectrum is projected onto a 1-dimensional linear detector array (as manufacturer information). The experiments were run in triplicate and carried out in five independent assays.

### Nano-indentation and micro-hardness tests

Resin-matrix composite specimens were embedded in autopolymerizing polyether-modified resin (Technovit 400™; Kulzer GmbH, Germany) in polyvinyl chloride mold [49]. Then, assemblies were cross-sectioned at 90° relative to the surface plane. Specimens were cross-sectioned by wet grinding on low speed using a standard laboratory metallographic machine (Struers, USA) and SiC papers ranging from 120 down to 400 mesh [49]. Cross-sectioned specimens were wet ground by using SiC paper from 300 down to 2400 mesh followed by polishing with 3000 and 6000 mesh (3-μm particle size) SiC paper (Trizact ™, 3M ESPE, USA). Then, specimens were ultrasonically cleaned in prophyl alcohol for 5 min and in distilled water for 10 min [50]. An optical micrography of a cross-sectioned specimen at × 200, is shown in Fig. 3F.

Nanoindentation tests were carried out with the loading axis perpendicularly to the specimen’s surfaces (Fig. 1). A nano-hardness tester (Nano Instruments, Inc. Knoxville; TN, USA) operated with a Berkovich diamond pyramid tip (apex angle of 143°) was used to make nanoscopic indentations in triplicate over the surfaces (*n* = 9). Load of 5 mN was applied at 0.04 mN/s onto the specimens for 15 s. Five points at different regions  were determined along the specimen. The shape function was determined by the Oliver and Pharr method (1992). Nanoindentation tests performed at 90° relative to the surface allow evaluating stress/strain fields induced during indentation across the specimen with respect to the displacement axis of the indenter. Nano-hardness and elastic modulus were acquired as a function of the position of the indenter axis relative to the specimens’ surface. Additionally, Vickers micro-hardness of the specimens was measured using a micro-indenter apparatus (Leica VMHT30™, Leica Microsystems, Germany) with a diamond pyramid-shape indenter. An indentation load at 200 g was applied onto the specimens for 20 s [44, 51, 52] at 5 different points on the surface of each specimen (*n* = 15). The plastic deformation of the indentation area was inspected using an optical microscope (Leica DM 2500 ™; Leica Microsystems, Germany) coupled to a computer for image processing by Leica Application Suite™ software.

### Chemical and microstructural analyses

At first, cross-sectioned specimens were inspected by optical microscopy at magnification ranging from × 30 up to × 1000. Microstructural analyses were performed using an optical microscope (Leica DM 2500 ™; Leica Microsystems, Germany) coupled to a computer for image processing, using Leica Application Suite™ software (Leica Microsystems, Germany). A number of five micrographs were acquired at different magnifications, for each specimen (*n* = 15). The software Adobe PhotoshopTM (Adobe Systems Software, Ireland) was used to analyze black and white images, considering the black region represented the organic matrix and the white regions represented the inorganic fillers. Image JTM software (National Institutes of Health, USA) was used to quantify the content of inorganic fillers. Then, surfaces were sputter coated with a AgPd thin layer for scanning electron microscopy (SEM) analyses by using a SEM unit JSM-6010 LV™ (JEOL, Japan) coupled to energy dispersive spectroscopy (EDS). The inorganic fillers and microstructure of the specimens were evaluated at high magnification ranging from × 1000 up to × 20,000 under (SE) secondary and (BSE) backscattered electrons, as shown in Fig. 4 [34–36]. Even though the chemical composition of the resin-matrix composites was provided by the manufacturers (in Table 1), elemental analyses were performed by energy dispersive spectroscopy (EDS) coupled to the SEM apparatus.

### Statistical analysis

Results were statistically analyzed by normality test Shapiro–Wilk and two-way ANOVA to determine statistical differences in the micro-hardness, nano-hardness, elastic modulus, and transmittance values between groups. The Student t-test was used to compare the results considering the content of inorganic fillers and polymerization time. A probability value  below 0.05 was considered significant. The power analysis performed by Student t-test or ANOVA to determine the number of specimens for each group (*n*) revealed a test power of 100% in the present study. Statistical analyses were carried out using Origin Lab statistical software (Origin Lab, Northampton, MA, USA).

## Results

### Chemical and microstructural analyses results

**Fig. 2:**
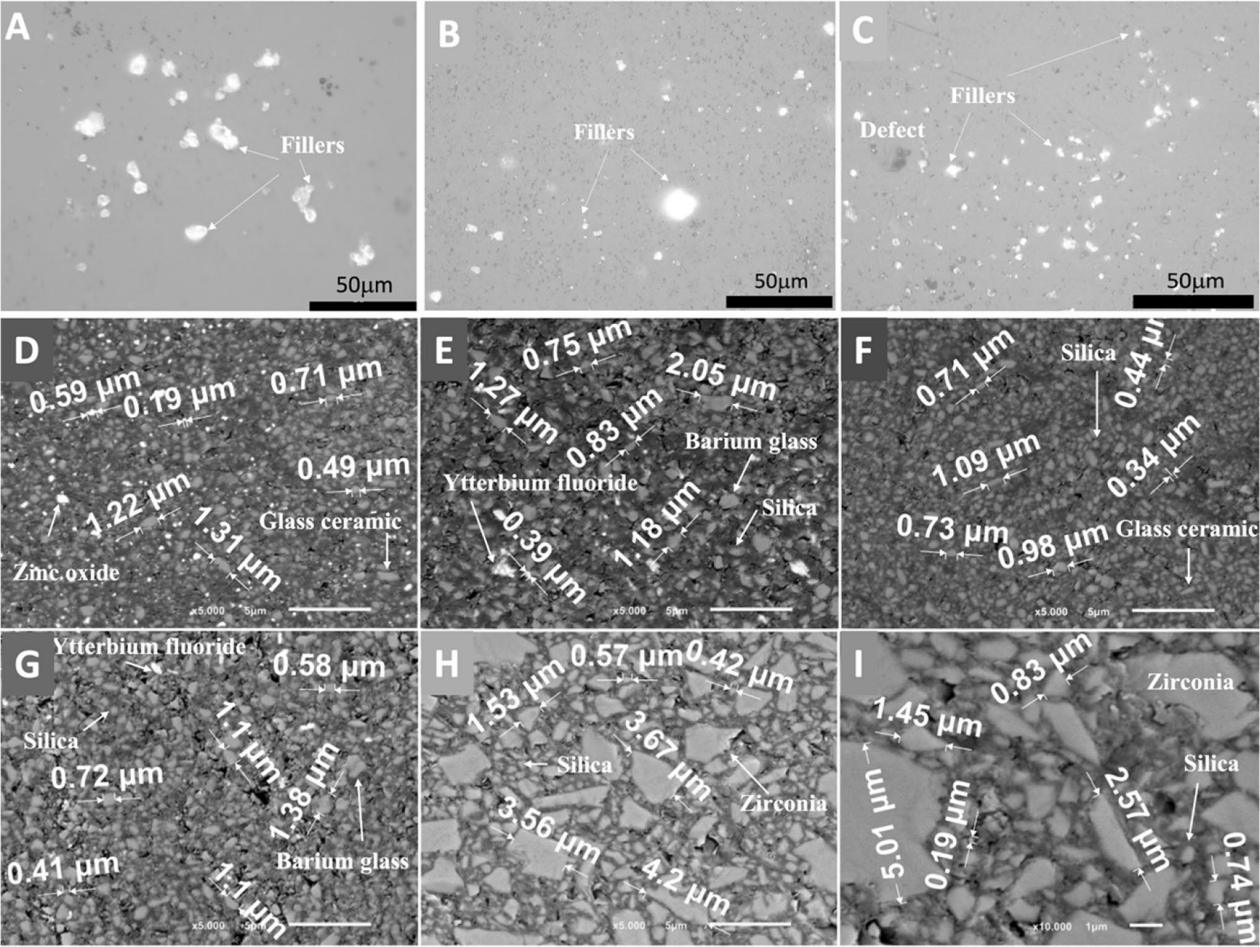
**A-C** Optical microscopy recorded at × 500 magnification for resin-matrix composites reinforced with **A** 60, **B** 78–80, and **C** 89 wt. % inorganic fillers. **D-I** SEM images recorded at × 5000 magnification for resin-matrix composites reinforced with **D** 60, **E** 62.5, **F** 74, **G** 78–80, and **H** 89 wt. % inorganic fillers. **I** SEM images recorded at × 10,000 magnification for resin-matrix composites reinforced with 89 wt. % inorganic fillers

The microstructure of the materials acquired by optical microscopy can be noticed in Fig. 2A–C. At low magnification, the white spots in the micrographs represent the micro-scale inorganic fillers with an irregular morphological aspect. The morphological aspects were evaluated at higher magnification by SEM at secondary or backscattered electrons mode, as seen in Fig. 2D–I.

On SEM analyses, the micro-scale inorganic fillers were clearly inspected and the sizes of the particles were measured for each material. The chemical analyses by EDS detected the presence of zinc oxide in the flowable resin-matrix composites reinforced with 60 wt. % inorganic fillers and ytterbium fluoride and silica for flowable resin-matrix composites reinforced with 62.5 wt. %. Traditional resin-matrix composites reinforced with 74 wt. % inorganic fillers revealed the presence of silica (spherical particles) and glass ceramic (with irregular shape). Traditional resin-matrix composites reinforced with 78–80 wt. % inorganic fillers showed the presence of spherical silica particles and glass ceramic with irregular shape.

The resin-matrix composite with 78–80 wt. % inorganic fillers revealed the presence of silica, ytterbium fluoride, and barium glass, while composites reinforced with 89 wt. % inorganic fillers revealed silica and zirconia in their microstructure. Chemical composition of the inorganic fillers corroborated with the technical information provided by the manufacturer (Table 1). As seen in Fig. 2I, the size of the zirconia particles ranged from 0.74 up to 5.01 μm.

### Optical transmittance analysis

**Fig. 3:**
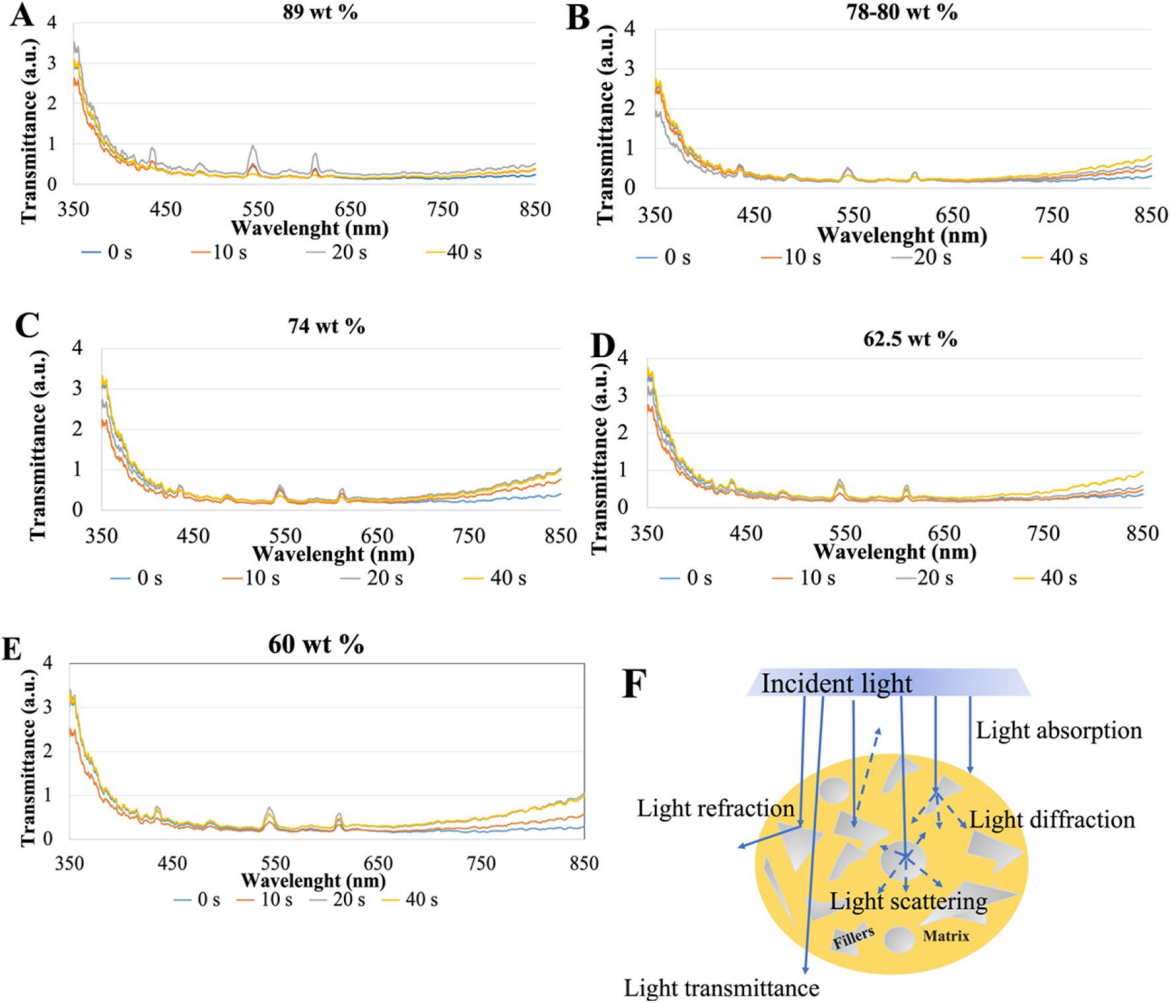
Transmittance (a.u.) of the resin-matrix composites in the wavelength range from 350 up to 850 nm at light irradiance for 0 s (non-polymerization), 10 s, 20 s, and 40 s. Resin-matrix composites containing **A** 89, **B** 78–80, **C** 74, **D** 62.5, and **E** 60 wt. % inorganic fillers. **F** Schematics of the light transmission through resin-matrix composites

As seen in Fig. 3, the transmittance spectra of non-polymerized specimens (0 s) were quite similar with a high transmittance within the ultraviolet (UV) region, below 400-nm wavelength.

The non-polymerized (np) specimens showed very low optical transmittance from 400- up 850-nm wavelength once the light was completely absorbed. Thus, non-polymerized resin-matrix composites showed no significant differences in optical transmittance for wavelengths above 450 nm (Fig. 3).

After the polymerization, there was an increase in optical transmittance of all specimens above 650-nm wavelength, i.e., when the optical spectrum approaches the near-infrared (near-IR) region. On higher polymerization time, the increase in the light transmittance was also higher, showing that the polymerization of the resin-matrix composites increases with the irradiance time. For instance, similar optical transmittance behavior was noticed for resin-matrix composites containing 62.5 and 78–80 wt. % inorganic fillers since an increase in transmittance occurred for polymerization time from 0 up to 40 s. Both resin-matrix composites contained the same types and quite similar dimensions of inorganic particles, as seen in Fig. 2.

Also, resin-matrix composites containing 60 and 74 wt. % inorganic fillers revealed similar light transmittance at irradiance for 20 or 40 s. The findings also suggested the light irradiance time can be performed for 20 s and therefore the clinical procedure validate such findings. Resin-matrix composites containing 60 and 74 wt. % inorganic fillers were composed of identical types of inorganic particles. Nevertheless, different light transmittances were recorded for resin-matrix composites reinforced with 89 wt. % inorganic fillers (Fig. 3A). A lower increase of light transmittance was detected for such composite for 20 or 40 s when compared to the other resin-matrix composites (Fig. 3B–E). Light transmittance was higher at irradiance time for 20 s compared to values recorded for 40 s. Statistical analyses of transmittance measurements at 850-nm wavelength are shown in Table 2.

**Table 2:** Analysis of variance of the transmittance results at 850-nm wavelength. Each measurement corresponds to an individual sample

Variation	Square sum	d.f	Square average	(*F*)	*p*
60 wt. % fillers for 10 s					
Between groups	0.182	1	0.182	116.139	< 0.001
Within groups	0.013	8	0.002		
Total	0.195	9			
60 wt. % fillers for 20 s					
Between groups	1.384	1	1.384	870.799	< 0.001
Within groups	0.013	8	0.002		
Total	1.397	9			
60 wt. % fillers for 40 s					
Between groups	1.174	1	1.174	668.013	< 0.001
Within groups	0.014	8	0.002		
Total	1.188	9			
62.5 wt. % fillers for 10 s					
Between groups	0.036	1	0.036	101.424	< 0.001
Within groups	0.003	8	0.000		
Total	0.038	9			
62.5 wt. % fillers for 20 s					
Between groups	0.137	1	0.137	386.916	< 0.001
Within groups	0.003	8	0.000		
Total	0.140	9			
62.5 wt. % fillers for 40 s					
Between groups	0.903	1	0.903	941.626	< 0.001
Within groups	0.008	8	0.001		
Total	0.910	9			
74 wt. % fillers for 10 s					
Between groups	0.337	1	0.337	258.886	< 0.001
Within groups	0.010	8	0.001		
Total	0.347	9			
74 wt. % fillers for 20 s					
Between groups	1.019	1	1.019	897.650	< 0.001
Within groups	0.009	8	0.001		
Total	1.028	9			
74 wt. % fillers for 40 s					
Between groups	0.877	1	0.877	255.582	< 0.001
Within groups					
Total					
78–80 wt. % fillers for 10 s					
Between groups	0.095	1	0.095	89.436	< 0.001
Within groups	0.008	8	0.001		
Total	0.103	9			
78–80 wt. % fillers for 20 s					
Between groups	0.241	1	0.241	21.852	0.002
Within groups	0.088	8	0.011		
Total	0.330	9			
78–80 wt. % fillers for 40 s					
Between groups	0.877	1	0.877	255.582	< 0.001
Within groups	0.027	8	0.003		
Total	0.905	9			
89 wt. % fillers for 10 s					
Between groups	0.046	1	0.046	77.409	< 0.001
Within groups	0.005	8	0.001		
Total	0.050	9			
89 wt. % fillers for 20 s					
Between groups	0.192	1	0.192	72.568	< 0.001
Within groups	0.021	8	0.003		
Total	0.213	9			
89 wt. % fillers for 40 s					
Between groups	0.061	1	0.061	22.891	0.001
Within groups	0.021	8	0.003		
Total	0.083	9			

**Fig. 4:**
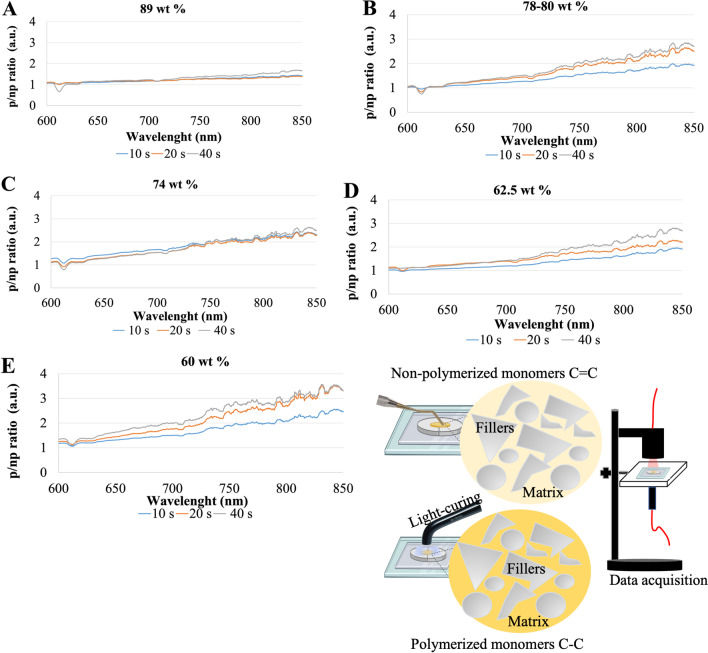
Transmittance ratio (a.u.) between the polymerized and non-polymerized resin-matrix composites in the wavelength range from 600 up to 850 nm at light irradiance. Resin-matrix composites containing **A** 89, **B** 78–80, **C** 74, **D** 62.5, and **E** 60 wt. % inorganic fillers. **F** Schematics of the light transmission through resin-matrix composites

**Fig. 5:**
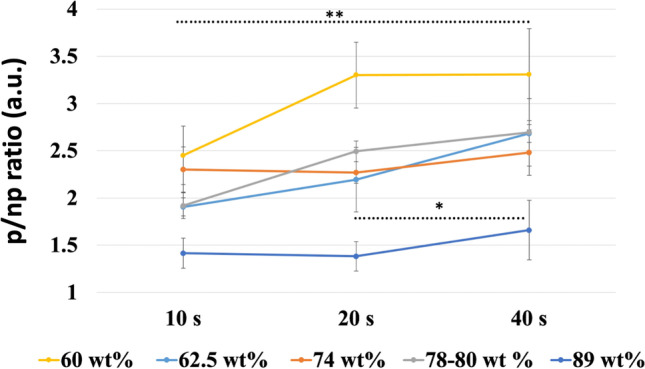
Ratio of the transmittance of the polymerized and non-polymerized (p/np ratio) resin-matrix composites for different light irradiance exposures. *Statistically significant (*p* < 0.05); **highly significant (*p* < 0.001)

In Fig. 4, the average ratio of the light transmittance between the polymerized (p) and non- polymerized (np) specimens (n/np ratio) is shown regarding the light wavelength range from 600 up to 850 nm for different polymerization time points: 10, 20, and 40 s. The results showed 1.6 times higher transmittance ratio for resin-matrix composites reinforced with 89 wt. % inorganic fillers under light irradiance at 850-nm wavelength in comparison with light irradiance at 650-nm wavelength (*p* = 0.0038). Also, p/np ratio was higher for 40 s compared to the ratio for 10 or 20s (Fig. 4A). The p/np ratio for resin-matrix composites reinforced with 78–80 wt. % inorganic fillers was higher under light irradiance for 20 s (*p* = 0.018) and 40 s (*p* = 0.0009) and lower for 10 s (*p* = 0.001), being 2.5 times higher at the 850-nm wavelength than at 600-nm wavelength (Fig. 4B). The p/np ratio for resin-matrix composites reinforced with 74 wt. % inorganic fillers was similar under light irradiance for 10 or 20 s (*p* < 0.0001), although p/np ratio was higher for 40 s (*p* = 0.0002) and increased × 2.5 from 600-nm up to 850-nm wavelength (Fig. 4C). Resin-matrix composites reinforced with 62.5 wt. % inorganic fillers showed a higher p/np ratio for 40 s (*p* < 0.0001) and lower p/np ratio for 10 or 20 s (*p* < 0.0005), but it was 2.5 times under light irradiance at 850-nm wavelength (Fig. 4D). At last, the resin-matrix composites reinforced with 60 wt. % inorganic fillers showed the highest p/np ratio since it was recorded at 3.5 times higher under light irradiance at 850-nm wavelength than that at 600-nm wavelength (*p* < 0.0001) (Figs. 4E and 5).

An overall comparison of the average ratio of the light transmittance between the polymerized and non-polymerized specimens is shown in Fig. 5. Statistical data on the p/np ratio at 850-nm wavelength are shown in Table 3.

**Table 3:** Analysis of variance of the p/np ratio at 850-nm wavelength for 10, 20, and 40 s compared with p/np ratio at 0 s

Variation	Square sum	d.f	Square average	(*F*)	*p*
p/np ratio on 60 wt. % fillers for 10 s					
Between groups	5.270	1	5.270	110.222	< 0.001
Within groups	0.383	8	0.048		
Total	5.653	9			
p/np ratio on 60 wt. % fillers for 20 s					
Between groups	13.244	1	13.244	217.447	< 0.001
Within groups	0.487	8	0.061		
Total	13.732	9			
p/np ratio on 60 wt. % fillers for 40 s					
Between groups	13.309	1	13.309	112.508	< 0.001
Within groups	0.946	8	0.118		
Total	14.256	9			
p/np ratio on 62.5 wt. % fillers for 10 s					
Between groups	4.242	1	4.242	148.963	< 0.001
Within groups	0.228	8	0.028		
Total	4.469	9			
p/np ratio on 62.5 wt. % fillers for 20 s					
Between groups	4.031	1	4.031	623.348	< 0.001
Within groups	0.052	8	0.006		
Total	4.083	9			
p/np ratio on 62.5 wt. % fillers for 40 s					
Between groups	7.086	1	7.086	1568.183	< 0.001
Within groups	0.036	8	0.005		
Total	7.122	9			
p/np ratio on 74 wt. % fillers for 10 s					
Between groups	4.242	1	4.242	148.963	< 0.001
Within groups	0.228	8	0.028		
Total	4.469	9			
p/np on 74 wt. % fillers for20 s					
Between groups	4.031	1	4.031	623.348	< 0.001
Within groups	0.052	8	0.006		
Total	4.083	9			
p/np ratio on 74 wt. % fillers for 40 s					
Between groups	5.485	1	5.485	188.508	< 0.001
Within groups	0.233	8	0.029		
Total	5.718	9			
p/np ratio on 78–80 wt. % fillers for 10 s					
Between groups	2.113	1	2.113	226.218	< 0.001
Within groups	0.075	8	0.009		
Total	2.187	9			
p/np ratio on 78–80 wt. % fillers for 20 s					
Between groups	5.583	1	5.583	934.345	< 0.001
Within groups	0.048	8	0.006		
Total	5.631	9			
p/np ratio on 78–80 wt. % fillers for 40 s					
Between groups	7.190	1	7.190	112.027	< 0.001
Within groups	0.513	8	0.064		
Total	7.703	9			
p/np ratio on 89 wt. % fillers for 10 s					
Between groups	0.433	1	0.433	34.296	< 0.001
Within groups	0.101	8	0.013		
Total	0.534	9			
p/np ratio on 89 wt. % fillers for 20 s					
Between groups	0.366	1	0.366	29.683	0.001
Within groups	0.099	8	0.012		
Total	0.465	9			
p/np ratio on 89 wt. % fillers for 40 s					
Between groups	1.092	1	1.092	21.784	0.002
Within groups	0.401	8	0.050		
Total	1.493	9			

### Mechanical behavior

**Fig. 6:**
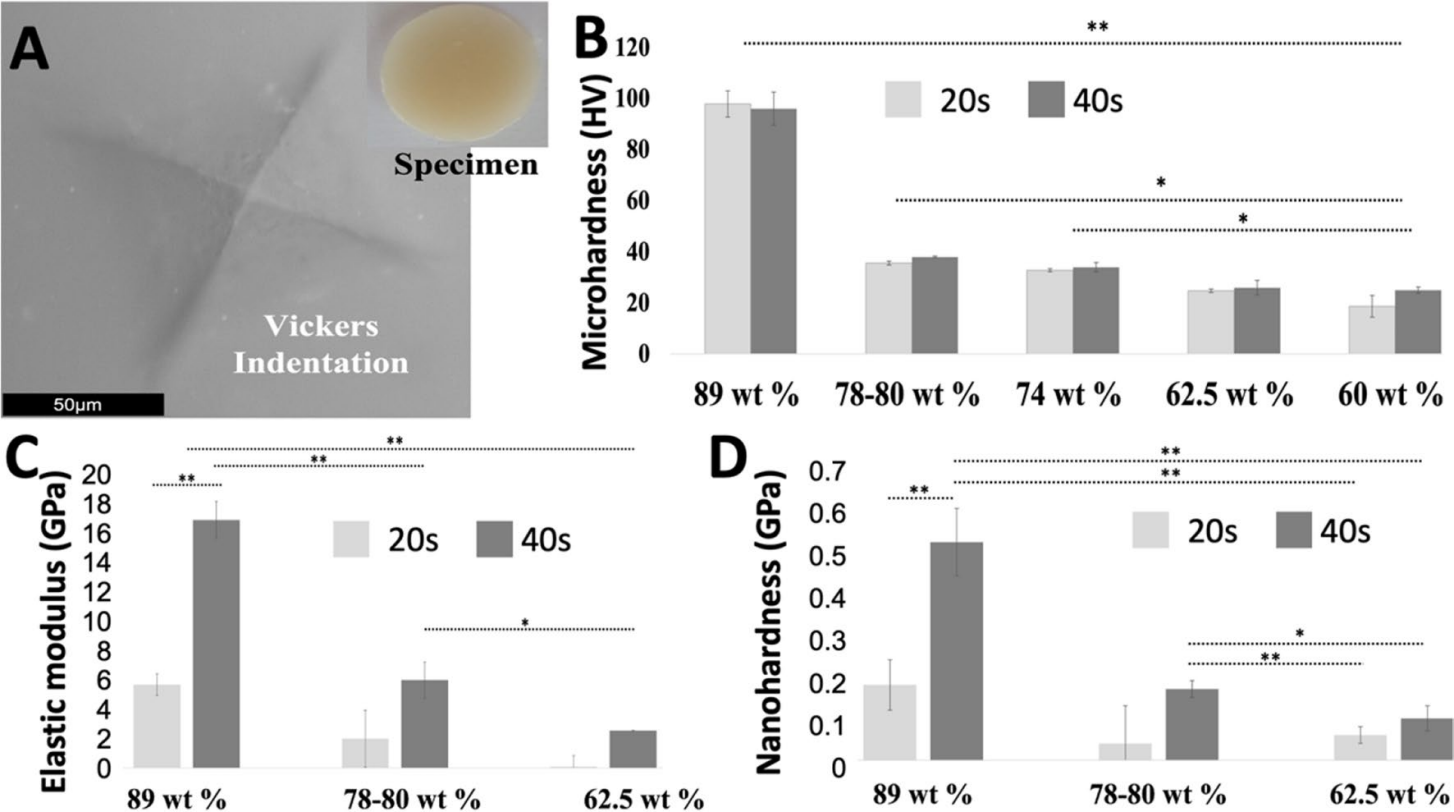
**A** SEM image of the Vickers indentation on the resin-matrix matrix specimen. Mean values of **B** micro-hardness, **C** elastic modulus, and **D** nano-hardness recorded for resin-matrix composites after polymerization for **A** 20 s or **B** 40 s. *Statistically significant (*p* < 0.05); **highly significant (*p* < 0.001)

In Fig. 6, the micro-hardness mean values are reported for the resin-matrix composites reinforced with different contents of inorganic fillers and polymerized under light irradiance for 20 or 40 s.

As expected, the highest microhardness mean values were recorded for resin-matrix composites reinforced with 89 wt. % inorganic fillers after polymerization for 20 or 40 s (*p* < 0.005). In the same way, resin-matrix composites reinforced with 78–80 wt. % inorganic fillers showed higher microhardness mean values than the other resin-matrix composites after polymerization for 20 s (*p* < 0.05). Flowable resin-matrix composites showed low micro-hardness values and the composites reinforced with 60 wt. % inorganic fillers revealed the lowest mean values.

Mean values of elastic modulus and nano-hardness of the materials recorded by nano-indentation assays are shown in Fig. 6C and D. After light irradiance for 20 or 40 s, the highest mean values of elastic modulus and nano-hardness were measured for resin-matrix composites reinforced with 89 wt. % (*p* < 0.001), followed by the composites reinforced with 78–80 wt. % (*p* < 0.05), while the lowest values were recorded for composites reinforced with 62.5 wt. %.

## Discussion

The present study focuses on the effect of inorganic particles on the light transmittance through resin-matrix composites by using visible and near-IR spectrophotometry. Thus, the findings acquired in this study revealed the percentage of inorganic fillers influences the light transmittance through the resin-matrix composites. Resin-matrix composites reinforced with 89 wt. % inorganic fillers showed a low light transmittance after polymerization for 10, 20, or 40s. Also, the highest content of fillers resulted in a low ratio of light transmittance on the resin-matrix composites regarding light absence before and light irradiance. On the other hand, flowable resin-matrix composites reinforced with 60 wt. % inorganic fillers revealed the highest light transmittance and consequently light transmittance ratio regarding light absence before and after light irradiance. In this way, the results validate the first hypothesis of the present study. The second hypothesis was rejected on the increase in the fillers’ amount inducing a high light-curing transmission through the material.

In this study, five types of resin-matrix composites were assessed considering the content of inorganic fillers, as seen in Table 1. One group of resin-matrix composites showed a high content of inorganic fillers at 89 wt. %, while another group revealed a low content of inorganic fillers at 60 wt. %. Inorganic fillers were properly identified by scanning electron microscopy coupled to energy dispersive spectroscopy. Resin-matrix composite reinforced with 89 wt. % inorganic fillers was composed of irregular shape glass fillers and spherical amorphous silica (Fig. 2). The dimensions of glass fillers were identified at micro-scale, while silica was detected at submicron- and nano-scale. Considering the type of inorganic fillers, a similar microstructure was noticeable on the resin-matrix composites reinforced with 62.5 or 80 wt. % inorganic fillers composed of ytterbium fluoride or amorphous silica. The mean size of most inorganic fillers was measured at submicron- and micro-scales although nano-scale silica was also enclosed. Furthermore, a similar microstructure was noticeable on the resin-matrix composites reinforced with 60 or 74 wt. % inorganic fillers composed of ytterbium fluoride or amorphous silica at micro-, submicron-, or nano-scale dimensions. The resin-matrix composites were selected considering a similarity of organic components as follows: Bis-GMA, Bis-EMA, TEGDMA, UDMA, HEMA (Table 1). The photoinitiator system, namely, camphorquinone, was analogous for each resin-matrix composite. Thus, the refractive index of monomers and inorganic fillers can affect the light transmittance through resin-matrix composites [1, 53, 54] that implies the light transmittance is highly dependent on the type of materials [4, 54, 55].

Low-light transmittance through resin-matrix composites reinforced with a high proportion of inorganic fillers (i.e., 89 wt. %) corroborates with the inorganic particles influencing the light distribution through the resin-matrix composites. Light transmittance increased with the content of inorganic fillers decreased down to 60 wt. %. Such findings are consistent with the literature data which indicates that increased amount of filler promotes lower transmittance values [1, 56, 57]. That was also noticed by the evaluation of the ratio between the transmittance of the polymerized and non-polymerized specimens since specimens with a high inorganic fillers’ content showed lower ratio values. Nevertheless, a lower ratio was detected for composites containing 62.5 wt. %, that can be explained by a significant proportion of opaque and irregular inorganic fillers, such as ytterbium fluoride [58]. After polymerization, the optical transmittance values were lower at UV region than the values recorded for the spectra infrared region. Light transmittance in the UV region for non-polymerized and polymerized specimens for 10, 20, or 40 s indicates that light is mainly absorbed in the visible light spectra, while UV light is mainly transmitted through the resin composites. It can be explained by the arrangement of the polymer networks after polymerization, which allows a proper and efficient light pathway [4, 56]. Camphorquinone consumption, which reduces light absorption, can also be a factor to increase the light transmittance [53]. Additionally, the exothermic nature of the polymerization chain reaction causes a transient decrease in the refractive index of resin-matrix composites due to a decrease in their density leading to an increase in the light transmittance [4, 56]. Thus, the decrease of the light transmittance in function of a high filler content can be expected although the nature of the inorganic fillers also plays a key role on the light transmission pathways. For instance, glass ceramics such as silica can provide a high translucency, while other glass ceramics provide a reflection of visible light. The size of inorganic particles also affected the light transmittance ratio since the light transmittance increased when the inorganic particles’ size decreased [4, 59]. A mixture of different inorganic fillers (i.e., glass ceramics and silica) at different sizes can be found in the chemical composition of current resin-matrix composites. In this way, a balance in the type and size of glass ceramics should be investigated in further studies.

The indirect evaluation of polymerization efficiency and relative DC percentage [29] can also be predicted by measuring the mechanical properties of the materials including hardness, strength, fracture toughness, or elastic modulus [60, 61]. Most of resin-matrix composite groups revealed higher light transmittance values after light irradiation for 40 s when compared to the values for 20 s. The results of ratio and light transmittance were consistent with the micro-hardness measurements, indicating a high polymerization of the materials after light curing for 40 s. However, commercially available resin-matrix composites can reveal varying optical behavior under light irradiation depending on several factors including exposure time. As seen in Figs. 4 and 5, the inorganic particles lead to light scattering that interferes in the light transmittance concerning a puzzling function of the filler size, distribution, and the mismatch of refractive indexes between the inorganic fillers and organic matrix [62, 63]. For instance, resin-matrix composites reinforced with 60 wt. % inorganic fillers were significantly dependent on the light exposure time in the present study. The transmittance ratio was lower under light exposure for 10 s when compared to the light irradiation for 20 or 40 s. Specimens containing 62.5 or 78–80 wt. % fillers showed a similar behavior regarding the ratio, although resin-matrix composites with 62.5 wt. % fillers were more dependent on light exposure time. On the other hand, the resin-matrix composite reinforced with 89 wt. % inorganic fillers was not dependent on the light exposure for 20 or 40 s. Vickers micro-hardness values showed slightly higher values for 20 s when compared to those recorded after polymerization for 40 s, which indicates the resin-matrix composite was properly polymerized for 20 s. The increased filler content of resin-matrix composites tends to improve mechanical properties of resin-matrix composites [58]. Micro-hardness values recorded for resin-matrix composites containing 89 wt. % and 78–80 wt. % inorganic fillers after polymerization for 20 and 40 s were similar to the results from previous studies [64]. Also, the elastic modulus and nano-hardness results were consistent with the values provided by the manufacturers. In this study, mechanical behavior of resin-matrix composites was highly dependent on the light exposure time, except on the Vicker’s microhardness for the resin-matrix composites containing 89 wt. % fillers. The highest values of Vicker’s microhardness recorded for the resin-matrix composite containing 89 wt. % fillers were resultant from the high inorganic fillers’ content. The elastic modulus values for resin composites with 89 wt. % fillers were in concordance with the values recorded in previous studies [31–33]. Also, the results showed differences in values recorded after light curing exposure over light curing for 20 or 40 s. The other groups of resin-matrix composites reinforced with 60, 62.5, and 78 wt.% fillers possess a higher portion of organic matrix, which requests a longer exposure of visible light for adequate polymerization. Then, the DC of monomers in the organic matrix of those resin-matrix composites is dependent on the light exposure time to achieve the required mechanical properties. The findings were validated considering the differences in light transmittance through the tested groups of resin-matrix composites with inorganic fillers ranging from 60 up 89 wt.%. Such findings are clinically relevant since an adequate polymerization via chair side light curing units must be accomplished by the clinicians to guarantee proper mechanical properties in the oral cavity environment [65]. Thus, the relationship among light irradiance, polymerization, DC, and inorganic fillers plays an important role on the physicochemical behavior of the resin-matrix composites. Commercially resin-matrix composites are progressively improved by manufacturers and a wide range of materials are available for restorative dentistry. Then, novel materials must be analyzed by traditional and alternative physicochemical approaches. The present data show that visible and near-IR spectrophotometry could be considered a potential alternative methodology to estimate the DC of resin-matrix composites through the evaluation of the light transmittance behavior. Considering the findings shown in the present study, further studies can be carried out involving a comparison of data with other methods such as Fourier transform infrared (FTIR) or near-infrared (IR).

## Conclusions

Within the limitations of the present in vitro study, the results indicate a decrease in the light transmittance through resin-matrix composites when the content of inorganic fillers increased in their chemical composition. However, the chemical composition, microstructure, and light irradiance exposure also varied concerning different commercially available resin-matrix composites. The concluding remarks can be drawn as follows:

- Glass ceramics, ytterbium fluoride, and amorphous silica were the inorganic fillers found within the tested resin-matrix materials. The size of inorganic fillers ranged from nano- up to micro-scale dimensions and the high content of micro-scale inorganic particles can change the light pathway and decrease the light transmittance through the materials. Also, inorganic fillers with a high light refractive index interfere in the light irradiance, and therefore a balance in the light refractive index between inorganic fillers and organic matrix plays a key role in the light transmittance through the materials.
- The lowest light transmittance values were recorded for a resin-matrix composite reinforced with 89 wt. % inorganic fillers, while the highest light transmittance was recorded on the resin-matrix composite reinforced with 60 wt. % inorganic fillers. However, a low light transmittance was noticed for the resin-matrix composite reinforced with 62.5 wt. % fillers due the presence of opaque inorganic fillers.
- The elastic modulus and hardness values of resin-matrix composites reinforced with 89 wt.% fillers (11 wt.% organic matrix) were not significantly affected by the variation in light exposure from 10 up 40 s. On the other side, resin composites reinforced with 60 wt. % fillers (40 wt.% organic matrix) showed low mechanical properties only after light irradiance for 10 s, and therefore their light transmittance ratio increased with the light exposure time, resulting in enhanced mechanical properties. That reveals a dependence of those composites to the light irradiance regarding the kinetics of degree of conversion of monomers in function of the proportion of the organic matrix.
- The present study data showed that visible and near-IR spectrophotometry could be considered a potential alternative methodology to estimate the DC of resin-matrix composites through the evaluation of the light transmittance behavior.

## Data Availability

Data will be available under request.

## References

[1] Masotti AS, Onófrio AB, Conceição EN, Spohr AM (2007). UV-vis spectrophotometric direct transmittance analysis of composite resins. Dent Mater.

[2] Klapdohr S, Moszner N (2005). New inorganic components for dental filling composites. Monatshefte Für Chemie/Chem Mon.

[3] Ferracane JL (2011). Resin composite–state of the art. Dent Mater.

[4] Balbinot E, Pereira M, Skupien JA, Balbinot CEA, da Rocha G, Vieira S (2019). Analysis of transmittance and degree of conversion of composite resins. Microsc Res Tech.

[5] Demarco FF, Corrêa MB, Cenci MS, Moraes RR, Opdam NJ (2012). Longevity of posterior composite restorations: not only a matter of materials. Dent Mater.

[6] Habib E, Wang R, Zhu XX (2017). Monodisperse silica-filled composite restoratives mechanical and light transmission properties. Dent Mater.

[7] Habib E, Wang R, Wang Y, Zhu M, Zhu XX (2016). Inorganic fillers for dental resin composites: present and future. ACS Biomater Sci Eng.

[8] Ruivo MA, Pacheco RR, Sebold M, Giannini M (2019). Surface roughness and filler particles characterization of resin-based composites. Microsc Res Tech.

[9] Elgendy H, Maia RR, Skiff F, Denehy G, Qian F (2019). Comparison of light propagation in dental tissues and nano-filled resin-based composite. Clin Oral Investig.

[10] Par M, Tarle Z, Hickel R, Ilie N (2018). Polymerization kinetics of experimental bioactive composites containing bioactive glass. J Dent.

[11] Leprince JG, Palin WM, Hadis MA, Devaux J, Leloup G (2013). Progress in dimethacrylate-based dental composite technology and curing efficiency. Dent Mater.

[12] Lopes-Rocha L, Ribeiro-Gonçalves L, Henriques B, Özcan M, Tiritan ME, Souza JCM (2021). An integrative review on the toxicity of bisphenol A (BPA) released from resin composites used in dentistry. J Biomed Mater Res B Appl Biomater.

[13] Fidalgo-Pereira R, Carpio D, Torres O, Carvalho O, Silva F, Henriques B (2022). The influence of inorganic fillers on the light transmission through resin-matrix composites during the light-curing procedure: an integrative review. Clin Oral Investig.

[14] Faria-E-Silva AL, Pfeifer CS (2017). Impact of thio-urethane additive and filler type on light-transmission and depth of polymerization of dental composites. Dent Mater.

[15] Pacheco RR, Carvalho AO, André CB, Ayres APA, de Sá RBC, Dias TM (2019). Effect of indirect restorative material and thickness on light transmission at different wavelengths. J Prosthodont Res.

[16] Fidalgo-Pereira R (2022) Relationship between the inorganic content and the polymerization of the organic matrix of resin composites for dentistry: a narrative review. RevSALUS 4(1). 10.51126/revsalus.v4i1.136

[17] Karabela MM, Sideridou ID (2011). Synthesis and study of properties of dental resin composites with different nanosilica particles size. Dent Mater.

[18] Xu T, Li X, Wang H, Zheng G, Yu G, Wang H (2020). Polymerization shrinkage kinetics and degree of conversion of resin composites. J Oral Sci.

[19] Kaisarly D, Gezawi ME (2016). Polymerization shrinkage assessment of dental resin composites: a literature review. Odontology.

[20] Rueggeberg FA, Giannini M, Arrais CAG, Price RBT (2017). Light curing in dentistry and clinical implications: a literature review. Braz Oral Res.

[21] Price RBT (2017). Light Curing in Dentistry. Dent Clin North Am.

[22] Stansbury JW (2000). Curing dental resins and composites by photopolymerization. J Esthet Dent.

[23] Stansbury JW (2012). Dimethacrylate network formation and polymer property evolution as determined by the selection of monomers and curing conditions. Dent Mater.

[24] Soto-Montero J, Nima G, Rueggeberg FA, Dias C, Giannini M (2020). Influence of multiple peak light-emitting-diode curing unit beam homogenization tips on microhardness of resin composites. Oper Dent.

[25] Carvalho Andrade K, PavesiPini NI, Dias Moda M, de Souza ESRF, Dos Santos PH, FragaBriso AL (2020). Influence of different light-curing units in surface roughness and gloss of resin composites for bleached teeth after challenges. J Mech Behav Biomed Mater.

[26] Santini A, Miletic V, Swift MD, Bradley M (2012). Degree of conversion and microhardness of TPO-containing resin-based composites cured by polywave and monowave LED units. J Dent.

[27] Fronza BM, Lewis S, Shah PK, Barros MD, Giannini M, Stansbury JW (2019). Modification of filler surface treatment of composite resins using alternative silanes and functional nanogels. Dent Mater.

[28] Fronza BM, Ayres A, Pacheco RR, Rueggeberg FA, Dias C, Giannini M (2017). Characterization of inorganic filler content, mechanical properties, and light transmission of bulk-fill resin composites. Oper Dent.

[29] Xu HH, Smith DT, Schumacher GE, Eichmiller FC, Antonucci JM (2000). Indentation modulus and hardness of whisker-reinforced heat-cured dental resin composites. Dent Mater.

[30] Gonçalves F, Azevedo CLN, Ferracane JL, Braga RR (2011). BisGMA/TEGDMA ratio and filler content effects on shrinkage stress. Dent Mater.

[31] Ilie N (2021). Microstructural dependence of mechanical properties and their relationship in modern resin-based composite materials. J Dent.

[32] Ilie N, Rencz A, Hickel R (2013). Investigations towards nano-hybrid resin-based composites. Clin Oral Investig.

[33] Randolph LD, Palin WM, Leloup G, Leprince JG (2016). Filler characteristics of modern dental resin composites and their influence on physico-mechanical properties. Dent Mater.

[34] Leprince J, Palin WM, Mullier T, Devaux J, Vreven J, Leloup G (2010). Investigating filler morphology and mechanical properties of new low-shrinkage resin composite types. J Oral Rehabil.

[35] Lucey SM, Santini A, Roebuck EM (2015). Degree of conversion of resin-based materials cured with dual-peak or single-peak LED light-curing units. Int J Paediatr Dent.

[36] Al-Zain AO, Eckert GJ, Lukic H, Megremis S, Platt JA (2019). Polymerization pattern characterization within a resin-based composite cured using different curing units at two distances. Clin Oral Investig.

[37] Issa Y, Watts DC, Boyd D, Price RB (2016). Effect of curing light emission spectrum on the nanohardness and elastic modulus of two bulk-fill resin composites. Dent Mater.

[38] Daugherty MMLWMM (2018). Effect of high- intensity curing lights on the polymerization of bulk-fll composites. Dent Mater.

[39] Par M, Marovic D, Attin T, Tarle Z, Tauböck TT (2020). Effect of rapid high-intensity light-curing on polymerization shrinkage properties of conventional and bulk-fill composites. J Dent.

[40] Pecho OE, Ghinea R, do Amaral EA, Cardona JC, Della Bona A, Pérez MM (2016). Relevant optical properties for direct restorative materials. Dent Mater.

[41] Chen M-H (2010). Update on dental nanocomposites. J Dent Res.

[42] Fugolin AP, Costa AR, Kono E, Quirk E, Ferracane JL, Pfeifer CS (2020) Influence of the organic matrix composition on the polymerization behavior and bulk properties of resin composites containing thiourethane-functionalized fillers. Eur Polym J 130. 10.1016/j.eurpolymj.2020.109664. 10.1016/j.eurpolymj.2020.109664 PMC7219823 32405085

[43] Goulart M, Fugolin AP, Lewis SH, Rodrigues JA, Erhardt MC, Pfeifer CS (2021). Thiourethane filler functionalization for dental resin composites: concentration-dependent effects on toughening, stress reduction and depth of cure. Mater Sci Eng C Mater Biol Appl.

[44] Colombo M, Gallo S, Poggio C, Ricaldone V, Arciola CR, Scribante A (2020) New resin-based bulk-fill composites: in vitro evaluation of micro-hardness and depth of cure as infection risk indexes. Materials (Basel) 13. 10.3390/ma13061308 10.3390/ma13061308 PMC7143874 32183115

[45] Moldovan M, Balazsi R, Soanca A, Roman A, Sarosi C, Prodan D et al (2019) Evaluation of the degree of conversion, residual monomers and mechanical properties of some light-cured dental resin composites. Materials (Basel) 12. 10.3390/ma12132109 10.3390/ma12132109 PMC6651104 31262014

[46] Faria-E-Silva AL, Dos Santos A, Girotto EM, Pfeifer CS (2019). Impact of thiourethane filler surface functionalization on composite properties. J Appl Polym Sci..

[47] Par M, Attin T, Tarle Z, Tauböck TT (2020) A new customized bioactive glass filler to functionalize resin composites: acid-neutralizing capability, degree of conversion, and apatite precipitation. J Clin Med 9. 10.3390/JCM9041173 10.3390/jcm9041173 PMC7230164 32325886

[48] Rode KM, Kawano Y, Turbino ML (2007). Evaluation of curing light distance on resin composite microhardness and polymerization. Oper Dent.

[49] Vahey BR, Sordi MB, Stanley K, Magini RS, Novaes de Oliveira AP, Fredel MC (2018). Mechanical integrity of cement- and screw-retained zirconium-lithium silicate glass-ceramic crowns to Morse taper implants. J Prosthet Dent.

[50] El-Safty S, Akhtar R, Silikas N, Watts DC (2012). Nanomechanical properties of dental resin-composites. Dent Mater.

[51] Poggio C, Lombardini M, Gaviati S, Chiesa M (2012). Evaluation of Vickers hardness and depth of cure of six composite resins photo-activated with different polymerization modes. J Conserv Dent.

[52] Jager S, Balthazard R, Dahoun A, Mortier E (2016). Filler content, surface microhardness, and rheological properties of various flowable resin composites. Oper Dent.

[53] Par M, Marovic D, Skenderovic H, Gamulin O, Klaric E, Tarle Z (2017). Light transmittance and polymerization kinetics of amorphous calcium phosphate composites. Clin Oral Investig.

[54] Fujita K, Nishiyama N, Nemoto K, Okada T, Ikemi T (2005). Effect of base monomer’s refractive index on curing depth and polymerization conversion of photo-cured resin composites. Dent Mater J.

[55] Fujita K, Ikemi T, Nishiyama N (2011). Effects of particle size of silica filler on polymerization conversion in a light-curing resin composite. Dent Mater.

[56] Howard B, Wilson ND, Newman SM, Pfeifer CS, Stansbury JW (2010). Relationships between conversion, temperature and optical properties during composite photopolymerization. Acta Biomater.

[57] Arikawa H, Fujii K, Kanie T, Inoue K (1998). Light transmittance characteristics of light-cured composite resins. Dent Mater.

[58] Amirouche-Korichi A, Mouzali M, Watts DC (2009). Effects of monomer ratios and highly radiopaque fillers on degree of conversion and shrinkage-strain of dental resin composites. Dent Mater.

[59] Arikawa H, Kanie T, Fujii K, Takahashi H, Ban S (2007). Effect of filler properties in composite resins on light transmittance characteristics and color. Dent Mater J.

[60] Ilie N, Hilton TJ, Heintze SD, Hickel R, Watts DC, Silikas N (2017). Academy of Dental Materials guidance-resin composites: part i-mechanical properties. Dent Mater.

[61] Ferracane JL, Hilton TJ, Stansbury JW, Watts DC, Silikas N, Ilie N (2017). Academy of Dental Materials guidance-Resin composites: Part II-Technique sensitivity (handling, polymerization, dimensional changes). Dent Mater.

[62] Par M, Spanovic N, Bjelovucic R, Skenderovic H, Gamulin O, Tarle Z (2018). Curing potential of experimental resin composites with systematically varying amount of bioactive glass: degree of conversion, light transmittance and depth of cure. J Dent.

[63] Aljabo A, Xia W, Liaqat S, Khan MA, Knowles JC, Ashley P (2015). Conversion, shrinkage, water sorption, flexural strength and modulus of re-mineralizing dental composites. Dent Mater.

[64] Lombardini M, Chiesa M, Scribante A, Colombo M, Poggio C (2012). Influence of polymerization time and depth of cure of resin composites determined by Vickers hardness. Dent Res J (Isfahan).

[65] de Mendonça BC, Soto-Montero JR, de Castro EF, Kury M, Cavalli V, Giannini M (2021). Effect of extended light activation and increment thickness on physical properties of conventional and bulk-filled resin-based composites. Clin Oral Investig.

